# Effects of infraglottal implantation on voice production in type I thyroplasty: a computational study

**DOI:** 10.1016/j.jbiomech.2026.113234

**Published:** 2026-02-27

**Authors:** Weili Jiang, Charles Farbos de Luzan, Liran Oren, Ephraim Gutmark, Xudong Zheng, Qian Xue

**Affiliations:** a Mechanical Engineering Department, Rochester Institute of Technology, Rochester, NY 14623, USA; b Department of Otolaryngology Head and Neck Surgery, University of Cincinnati, Cincinnati, OH 45267, USA; c Department of Aerospace Engineering and Engineering Mechanics, University of Cincinnati, Cincinnati, OH 45267, USA

**Keywords:** Vocal fold biomechanics, Voice production, Fluid–structure interaction, type Ithyroplasty, Virtual surgery

## Abstract

Type I thyroplasty (TT1) is one of the primary phonosurgical interventions for unilateral vocal fold paralysis. This study investigated the effects of infraglottal implant placement in TT1 on voice production using numerical simulations. The laryngeal and implant geometries were reconstructed from computed tomography scan of a canine larynx with a hand-carved Silastic implant. Fluid–structure interaction simulations, coupling the continuum finite element vocal fold and implant models with a one-dimensional Bernoulli-based glottal flow model, were conducted to evaluate vocal fold vibration across a range of implant stiffnesses, insertion depths, and vertical positions. Effects of implant parameters on pre-phonatory shape and stiffness, glottal flow and vocal fold dynamics and acoustic outcome have been analyzed. The results revealed that infraglottal implants can enhance both sound pressure level (SPL) and vocal efficiency (VE) when the insertion depth exceeds a certain threshold. The observed enhancement in SPL and VE was attributed to an increased vertical stiffness gradient in the vocal folds induced by the inferior implant, which leads to greater force asymmetry between vocal folds opening and closing, thereby promoting more effective energy transfer from the glottal airflow to the vocal folds. The result is larger vocal fold vibrations, which leads to a larger SPL and may also contribute to the improved VE. The current simulation is based on the simulation of one single canine larynx. Future studies incorporating more subjects would help to generalize the current findings.

## 1. Introduction

During human voice production, vocal folds oscillations transform steady airflow into pulsatile flow, serving as the primary acoustic source. Unilateral vocal fold paralysis (UVFP)—caused by disrupted neural input to one vocal fold—results in glottic insufficiency and asymmetric vibration, leading to a breathy, weak voice and vocal fatigue (Liu et al., 2014; Rubin and Sataloff, 2007; Schwarz et al., 2006). Type I thyroplasty (TT1) (Isshiki et al., 1974) is the primary surgical intervention for UVFP, involving the insertion of a medialization implant to improve glottic closure and restore vibratory function. The optimal implant configuration varies with individual laryngeal anatomy and is often determined intraoperatively through voice assessment and a trial-and-error approach.

Recently, clinical reports have suggested that placing the implant in an infraglottal (inferior) position, rather than the standard mid-membranous site, may lead to better voice outcomes (Desuter et al., 2020; Laccourreye et al., 2021). Supporting this observation, subsequent experimental studies using ex vivo human and canine larynges (Cohen et al., 2024; Oren et al., 2024; Zhang and Chhetri, 2019) have also demonstrated enhanced voice outcomes with infraglottal implant. Several mechanisms have been proposed. Zhang and Chhetri (2019) showed in ex vivo human larynges that infraglottal implant improves glottal closure and enhances acoustic measures (e.g., higher-order harmonics and cepstral peak prominence (CPP)) compared to more superior medialization at the same medialization level. They attributed this improvement to increased effective vocal fold thickness. However, in a subsequent study (Cameron et al., 2020), they found that stiffer implants, while also increasing medial surface thickness, did not yield clear acoustic benefits, suggesting that vertical thickness alone may not fully explain voice improvements. Complementing these findings, Farbos De Luzan et al. (2025) compared implant placement positions in excised canine larynges and found that, although infraglottal implants produced smaller pre-phonatory vertical thickness than standard glottal implants, they generated greater dynamic thickness during vibration. This promoted more effective mucosal wave propagation and larger divergent angles during glottis closing, which have been associated with improved vocal efficiency (VE) (Farbos de Luzan et al., 2021a; Kirsh et al., 2017; Michaud-Dorko et al., 2024b). Together, these studies highlight that improved voice outcomes from infraglottal placement likely arise from changes in vibratory dynamics governed by fluid–structure interaction (FSI), rather than from pre-phonatory geometry alone.

Another factor that may contribute to the improved voice outcomes with infraglottal placement is the vertical stiffness gradient (VSG) of the vocal fold. VSG, characterized by decreasing stiffness from the inferior to superior aspect, has been observed in both human (Chhetri et al., 2011; Michaud-Dorko et al., 2024a) and canine (Cohen et al., 2024; Michaud-Dorko et al., 2024a; Oren et al., 2014a) vocal folds. Cohen et al. (2024) measured VSG in seven excised canine larynges under three conditions, including no implant, glottal-level implant, and infraglottal implant, and found that infraglottal implant increased VSG, whereas glottal-level implant merely increased overall vocal fold stiffness without enhancing the gradient. Using a three-dimensional (3D) finite element model, Geng et al. (2016) found that greater VSG increases vertical phase difference and glottal divergent angle during vibration, leading to higher peak flow rate, larger closed quotient, higher sound pressure level (SPL), and reduced phonation threshold pressure. Consistent with these findings, Cohen et al. (2024) observed higher VE in excised canine larynges with infraglottal implants. Studies by the same group (Farbos de Luzan et al., 2021a; Oren et al., 2024), also using excised canine larynx experiments, further showed that larger VSG promotes greater divergent angle during glottis closing. Oren et al. (2024) hypothesized that a larger divergent angle promotes flow-separation vortices (FSV), which reduces negative intraglottal pressure and facilitates vocal fold closing (Farbos de Luzan et al., 2021b; Jiang et al., 2023; Khosla et al., 2009, 2008, 2007; Mihaescu et al., 2010). In their experiments, infraglottal implants, compared with glottal-level implants, significantly enhanced glottal divergence angles, generated stronger FSVs, and increased VE, although no statistically significant change in CPP was observed.

In summary, multiple studies have reported benefits of infraglottal implants, largely attributed to increased vertical thickness and enhanced VSG. Reported outcomes, however, vary across studies. Building on these findings, this study investigates how implantation vertical location, insertion depth, and material properties collectively influence vocal fold biomechanics and voice outcomes. A subject-specific canine laryngeal model is employed, with material properties determined using microindentation measurements at different anterior-posterior and superior-inferior positions, enabling accurate characterization of VSG. FSI simulations with virtual implant insertion are performed (Movahhedi et al., 2021), coupling finite element models of the vocal folds and implant with a one-dimensional (1D) Bernoulli’s equation-based glottal flow model. This framework enables systematic quantification of the effect of implant location under various stiffness and insertion depth conditions. The causal relationships among pre-phonatory medial surface shape, VSG, and voice outcomes are analysed.

## 2. Methods

**Fig. 1.**
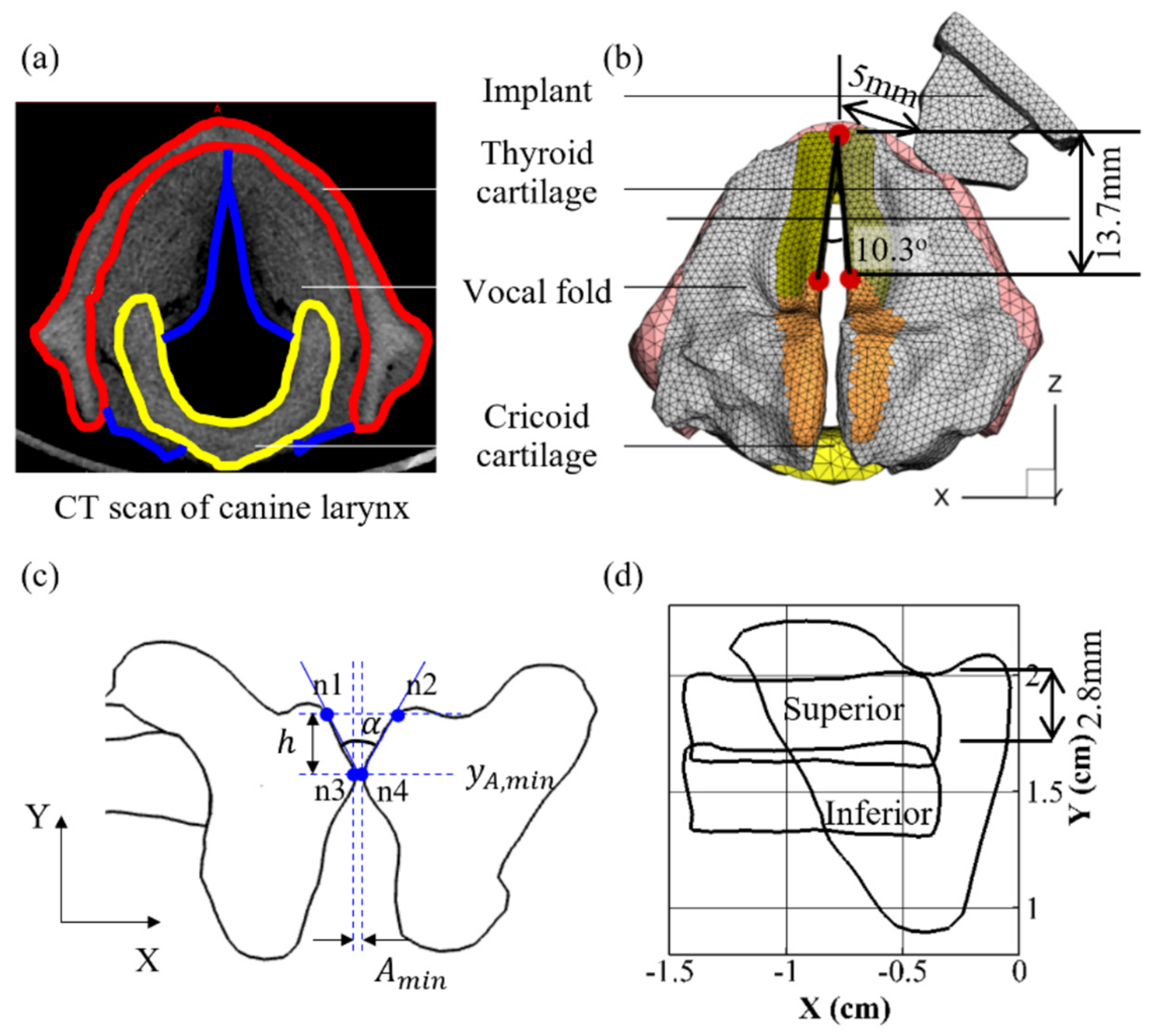
Geometry reconstructed from the CT scan: (a) The CT scan showing a transverse plane with cartilages and tissue segmented; (b) the finite element model of the larynx including the two-layer vocal folds, the cartilages and the implant; (c) Definition of key parameters related to vocal fold dynamics. *A_min_* and *y_A,min_* are the glottal area and its axial location, respectively. n1 – n4 are four marker points defined based on the superior edge of the vocal folds and *A_min_* in the mid-coronal plane. *h* is the glottal divergence height. *α* is the glottal angle; (d) the vertical locations of the implants at the most superior (Loc = 0.5 mm) and most inferior (Loc = 3.5 mm) locations.

An excised canine larynx and a TT1 implant were reconstructed from computed tomography (CT) scans using the commercial software Mimics 16.0 (Materialise, Leuven, Belgium) (Fig. 1a). Fig. 1b and Fig. 1c show the reconstructed larynx and implant models and the definition of key parameters related to vocal fold dynamics, respectively. Both the cover and body layers of the vocal folds were modeled using a fiber-reinforced material formulation (Geng et al., 2020; Pham et al., 2018) with VSG applied in the cover layer. Neo-Hookean material model was used to describe the material properties of all cartilages, the paraglottic space and the implant.

UVFP was reproduced by adducting the healthy fold through applying a medial force on the vocal process, while the paralyzed fold remained at rest (Oren et al. 2024). The paralyzed fold was adducted by the insertion of an implant to generate the pre-phonatory posture. Active muscle force was not included. Following implantation, FSI simulations were conducted using the deformed vocal fold and implant. The finite element model of the vocal fold and implant was coupled with a 1D reduced-order glottal flow model based on the Bernoulli equation (Geng et al., 2021). Penalization-based contact model was employed between the medial surface of the implant and the lateral surface of the paraglottic layer, and between the two sides of the vocal folds to prevent penetration during pre-phonatory posture and FSI. A constant subglottal pressure of 1.5 kPa was applied in all cases. Pressure calculated from Bernoulli’s equation was applied from the subglottal region to the location of the minimum glottal area. Downstream of this point, zerogauge pressure was assumed. The implanťs vertical location (Loc), material stiffness (S), and insertion depth (D) were systematically varied as listed in Table 1.

Acoustic parameters including SPL, CPP, VE were calculated based on the glottal flow rate. Specifically, sound pressure (*p*^ʹ^) was calculated as proportional to the time derivative of the glottal flow rate (Lighthill, 1978). SPL and CPP were calculated based on *p*^ʹ^. VE was calculated as the ratio of the acoustic power to aerodynamic power. To assess the effect of vocal fold adduction, pre-phonatory contact area was calculated as the integrated area along the vocal folds where the inter-fold distance was below 0.01 mm; VSG was calculated from indentation-measured stiffness as the ratio of the difference between inferior and superior medial-surface stiffness to their sum. Pearson correlation coefficient (r) was computed to quantify the linear correlation between different parameters. Correlations were classified as strong with |r| >0.7 (Mukaka, 2012). Correlations were considered statistically significant when p value was less than 0.05.

Several other key statistical quantities related to the vocal fold and glottal flow dynamics were computed. More details of Materials and Methods are provided in the supplementary material.

## 3. Results and discussion

Out of all the simulation cases, three failed to converge during FSI simulation: stiffness S3 with Loc = 0.5 and 1.0 mm at D = 1.5 mm, and Loc = 0.5 mm at D = 1.0 mm, corresponding to the stiffest implant inserted in the deepest and most superior locations, where excessive deformation occurred around the superior-anterior location of the vocal fold. All remaining cases completed successfully with simulations running for a minimum of 0.3 s. Subsequent analyses were based on these converged cases.

### 3.1. Effect of implant parameters on acoustic outcomes

**Fig. 2.**
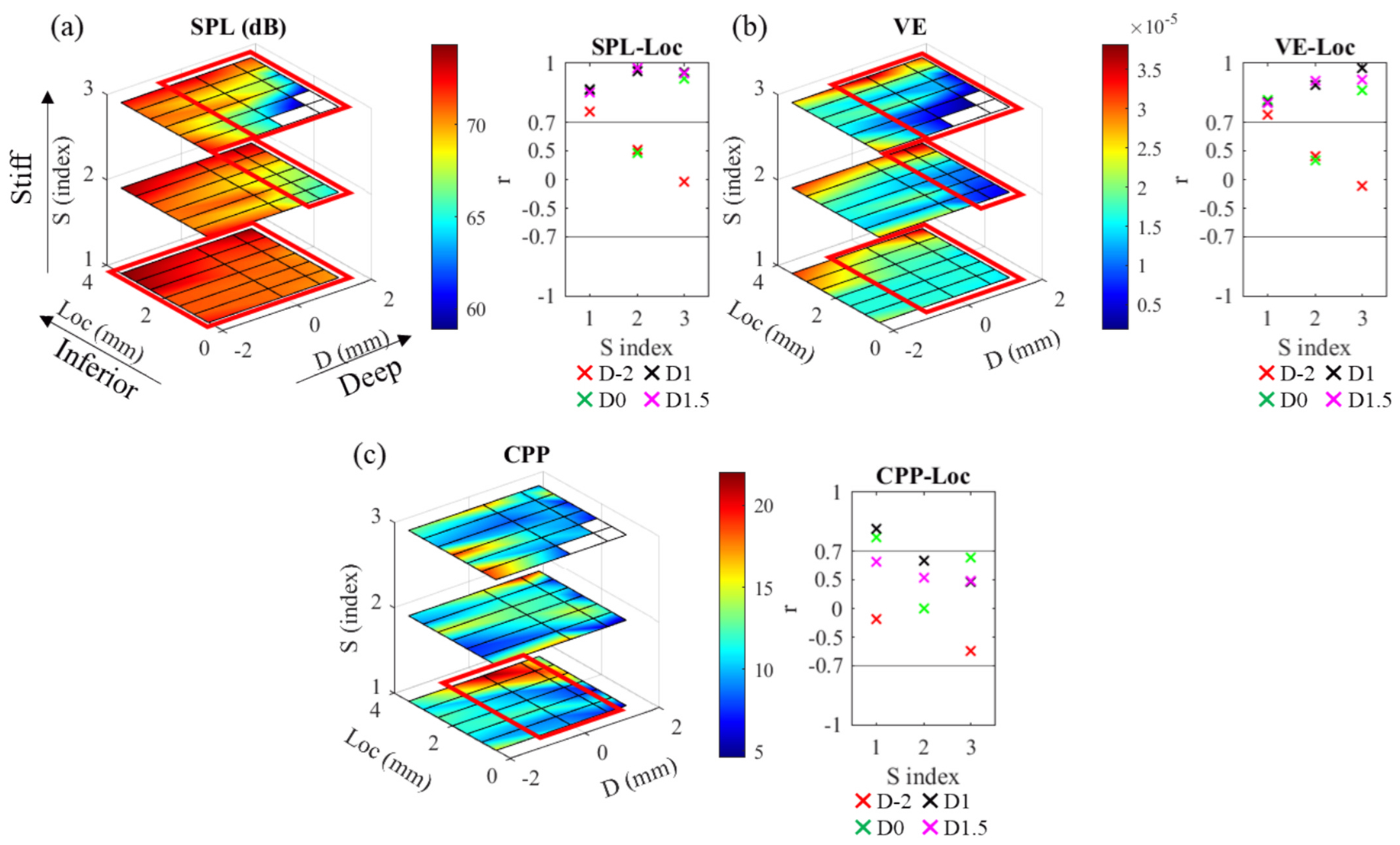
The acoustic outcomes —(a) SPL, (b) VE, and (c) CPP — as functions of implant vertical location (Loc), insertion depth (D), and stiffness (S). For each parameter, the left panel show contour plots of the simulated results, while the right panel display Pearson correlation coefficients (r) between vertical location and the corresponding acoustic measure for different stiffness–depth combinations. Red solid shapes denote the stiff-depth conditions manifesting strong, significant correlation between the corresponding parameter and the vertical location. The horizontal lines denote |r|=0.7, the threshold for a strong correlation. (For interpretation of the references to colour in this figure legend, the reader is referred to the web version of this article.)

For SPL (Fig. 2a), values ranged from 59 to 74 dB across all cases. The lowest SPL (59 dB) occurred with a stiff implant inserted deeply at a superior location, while the highest SPL was produced by a soft implant placed inferiorly. A strong positive correlation between SPL and vertical location was observed in all S1 cases, in S2 cases with D ≥ 1.0 mm, and in S3 cases with D ≥ 0.0 mm (red solid symbols in Fig. 2a). Overall, moving the implant from a superior to an inferior location significantly increases SPL, with greater sensitivity observed in stiffer implants, consistent with the trend reported by Zhang et al. (2015).

For VE (Fig. 2b), values were generally higher when the implant was placed at more inferior locations across all stiffness and depth conditions. VE decreased notably with superior, deeper insertion, particularly under higher stiffness. A strong positive correlation between VE and vertical location was observed in cases marked by red solid symbols in Fig. 2b. Overall, moving the implant from a superior to an inferior position significantly increases VE, consistent with the SPL trend in Fig. 2a and excised model observations (Oren et al., 2024).

CPP (Fig. 2c) ranged from 4.6 to 22 dB, consistent with previously reported values for both normal and dysphonic voice conditions (Buckley et al., 2023; Murton et al., 2020). Unlike SPL and VE, CPP did not exhibit a consistent dependence on implant vertical location across the range of stiffness and depth combinations that we investigated.

These results indicate that inferior implant enhances SPL and VE, and a minimum degree of medialization is necessary before the effects of implant positioning on acoustic outcomes can manifest, consistent with observations in Orestes et al. (2014). Hereafter, cases showing a strong correlation between vertical location and both SPL and VE are referred as to the group of interest, specifically, cases with D ≥ 0.0 mm in S1 and S3, and D ≥ 1.0 mm in S2 (red solid symbols in Fig. 2b).

### 3.2. Effect of implant parameters on glottal flow and vocal fold dynamics

**Fig. 3.**
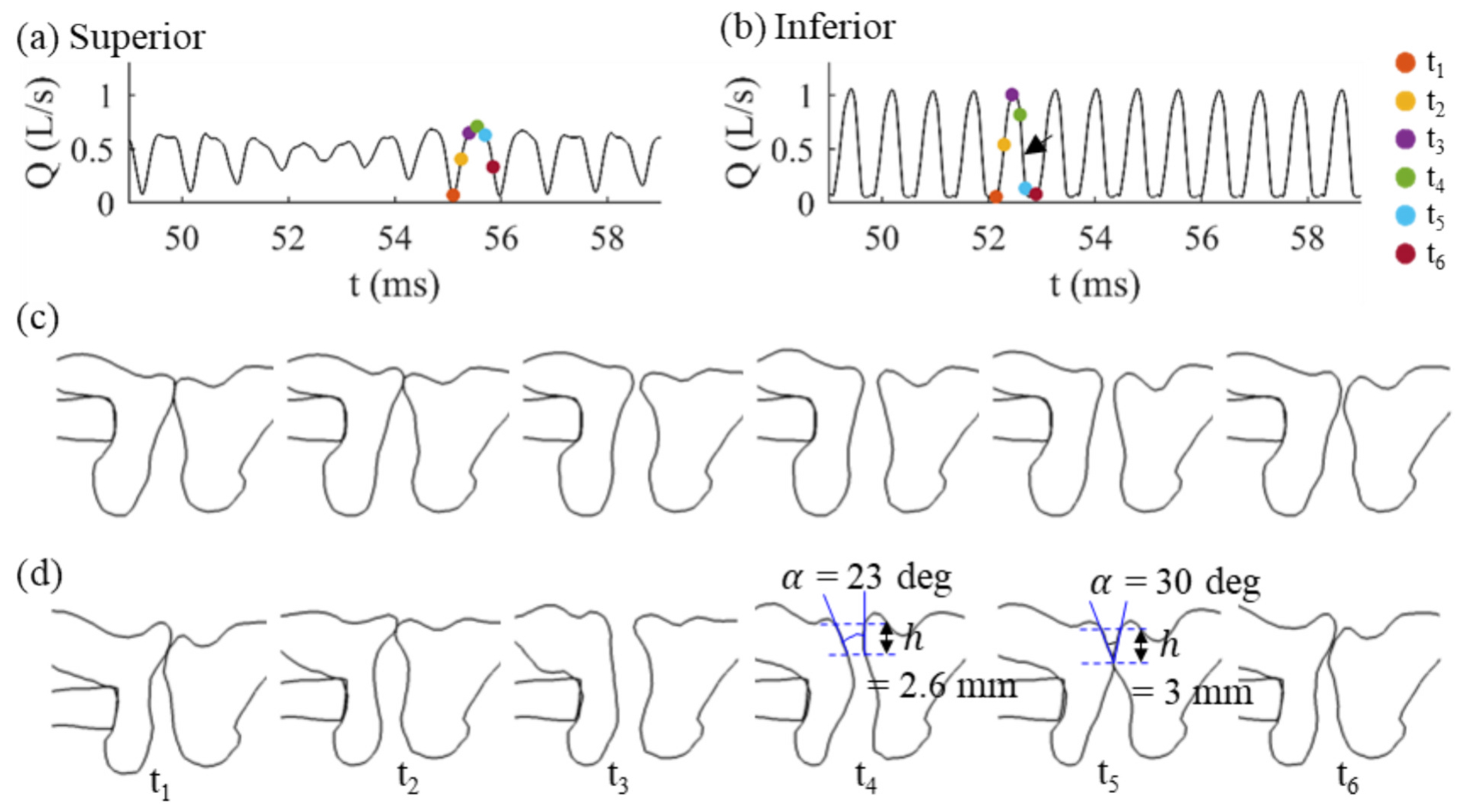
Glottal flow rate and vocal fold mid-coronal profiles in D = 0.0 mm, S3 (1386 kPa) condition: (a) Flow rate at superior insertion (Loc = 0.5 mm) and (b) inferior insertion (Loc = 0.5 mm). (c), (d) show the mid-coronal profiles of the vocal folds corresponding to the six consecutive time instants with 0.15 ms intervals, denoted in (a), (b), respectively. The instant of t_5_ in (d) has been adjusted (now the time interval between t_4_ and t_5_ is 0.1 ms) to the time instant corresponding to the maximum divergent angle. The measurement of the divergent angle (*α*) and divergent height (*h*) are denoted in the subfigure for t_4_ and t_5_. The arrow in (b) denotes the MFDR instant.

Two representative cases were selected from the group of interest under the conditions of D = 0.0 mm and S3, with the implant placed at a superior location and an inferior location. Fig. 3 compares the glottal flow rate and vocal fold dynamics between the two cases. A small opening persisted at the posterior end of the vocal folds even when most of the folds were closed, preventing the glottal flow from reaching zero. Therefore, the vocal folds were considered closed during periods when the glottal flow rate fell below a threshold value corresponding to that residual opening.

When the implant was positioned superiorly (Fig. 3a), the glottal flow waveform showed incomplete closure, with significant cycle-to-cycle variation. The closed quotient was 0 and the skewness quotient was 0.89. The vocal folds showed a convergent shape throughout most of the vibration cycle (Fig. 3c). The closed phase was brief and occurred mainly near the superior margin. This vibration pattern suggests no significant vertical phase difference or mucosal wave propagation. Furthermore, the left and right vocal folds exhibited almost symmetric motion, with similar vibratory patterns.

In contrast, when the implant was placed inferiorly (Fig. 3b), the glottal flow waveform became highly periodic. Complete glottal closure was achieved during each cycle with a closed quotient of 0.1 and a skewness quotient of 1.36. The vocal fold dynamics (Fig. 3d) revealed pronounced left–right asymmetry, with stronger vibration from the paralyzed vocal fold (left side). At the onset of the opening phase (t1), the superior portion of the left fold crossed the midline, pushing the healthy fold toward the right. As the opening continued (t_2_), both folds began to separate starting from their inferior aspects. At peak flow (t_3_), the left (implanted) vocal fold exhibited a relatively straight medial profile, while the right (healthy) maintained a convergent shape. During the closing phase (t_4_, t_5_), the left fold developed a strong divergent profile. The glottal divergence continued to increase during the closing phase, peaking at the end of the closing phase (t_5_), with a divergent angle of 30 degrees and a divergent height of 3 mm. The initial closure is approximately symmetric on the two sides. However, at late closure (t_6_), the healthy side was pushed laterally. The instant of MFDR occurred between t_4_ and t_5_ (black arrow in Fig. 3b). A similar observation has been reported in our previous study (Jiang et al., 2023) that the divergent angle continued increasing during closing and MFDR occurred around mid-closing.

Similar behaviors were observed across the group of interest. Specifically, inferior implant promoted more pronounced convergent-divergent motion of the vocal folds, leading to more complete glottal closure, a longer closed phase, and a more periodic flow waveform. In contrast, superior implant generally led to reduced vibration amplitude, incomplete closure, and greater cycle-to-cycle variability. These patterns were not evident in cases outside the group of interest.

**Fig. 4.**
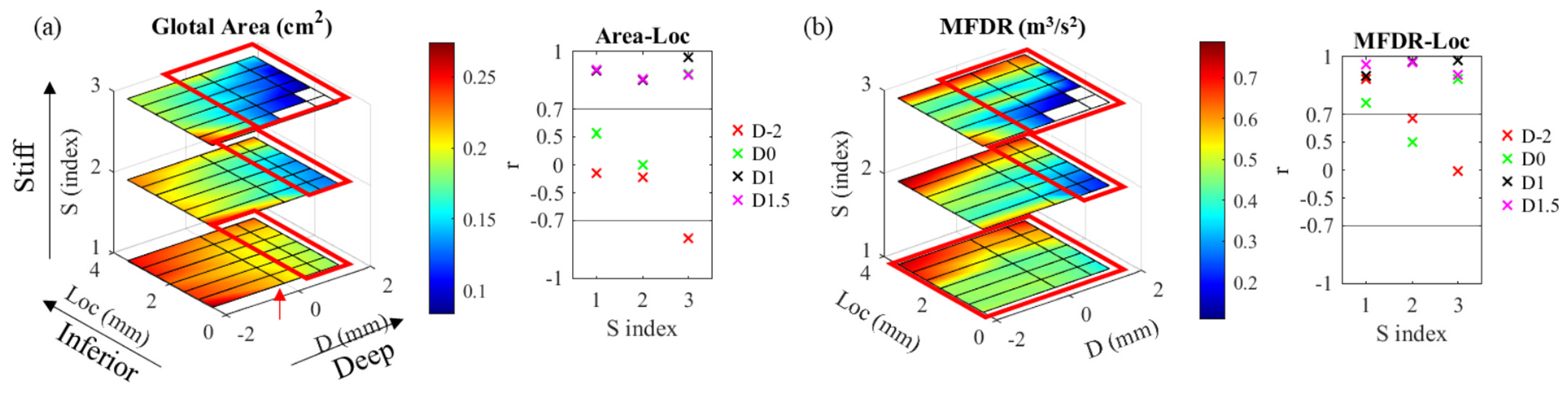
Glottal area related parameters: (a) maximum glottal area and (b) the maximum flow declination rate (MFDR). In each panel, it shows the contour of the corresponding parameter, and the Pearson correlation coefficient (r) between the corresponding parameter and the vertical location in each implant stiffness- depth combination. The figure format is the same with Fig. 2. Red solid shapes denote the stiff-depth conditions manifesting strong, significant correlation between the corresponding parameter and the vertical location. The red arrow denoted the S1, D = 0.0 mm condition with r = 0.54, p = 0.22. (For interpretation of the references to colour in this figure legend, the reader is referred to the web version of this article.)

Fig. 4 presents two key glottal flow-related parameters — maximum glottal area and MFDR — as functions of implant vertical location, insertion depth, and stiffness. Most cases within the group of interest showed a strong positive correlation between vertical location and maximum glottal area, except for the case of S1, D = 0.0 mm, where the correlation was moderate (r = 0.54; red arrow in Fig. 4a). For MFDR (Fig. 4b), all cases within the group of interest exhibited a significant positive correlation with vertical location. MFDR, which closely relates to sound intensity (Titze, 2006), also showed a strong correlation with SPL (r = 0.91, p < 0.001). MFDR also varied more strongly with vertical location for stiffer implants, consistent with the corresponding SPL trends. Overall, the results showed that, within the group of interest, inferior implant increased vibration amplitude and MFDR.

### 3.3. Effect of implant parameters on pre-phonatory shape and stiffness

**Fig. 5.**
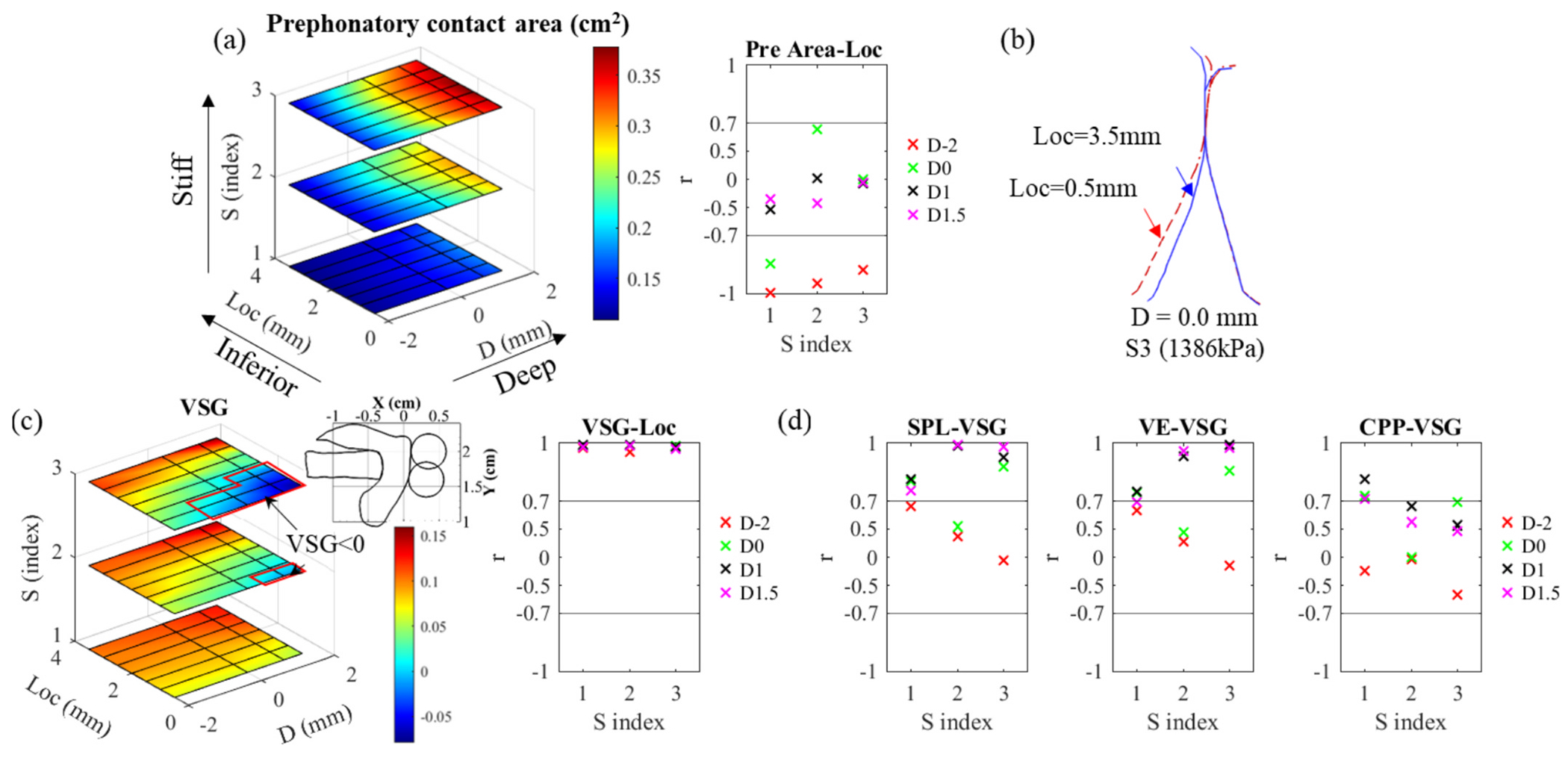
(a) Pre-phonatory contact area as a function of implant vertical location (Loc), insertion depth (D), and stiffness (S). Pearson correlation coefficients (r) between vertical location and the pre-phonatory contact area for each stiffness–depth combination are shown in the right panels. (b) Comparison of pre-phonatory vocal fold profiles at the mid-coronal plane for S3, D = 0.0 mm, at Loc = 3.5 mm and 0.5 mm. (c) VSG as a function of vertical location, insertion depth, and stiffness, along with corresponding Pearson correlation coefficients. (d) Pearson correlation coefficients between VSG and three acoustic parameters: SPL, VE, and CPP.

Fig. 5a shows that the pre-phonatory contact area was primarily affected by insertion depth and implant stiffness, with minimal dependence on vertical location. Within the group of interest, the correlation coefficient between vertical location and pre-phonatory contact area was |r|<0.52, *p >* 0.24 (except D = 0.0 mm, S1).

Fig. 5b shows mid-coronal vocal fold profiles for two representative cases (S3, D = 0.0 mm) at Loc = 0.5 mm and Loc = 3.5 mm. Despite the difference in vertical placement, both implants effectively closed the glottal gap, resulting in similar pre-phonatory contact areas. The superior implant, however, produced greater left–right asymmetry, whereas the inferior implant primarily displaced the lower portion of the fold, creating a more symmetric pre-phonatory configuration. Interestingly, the vibration pattern that followed was more asymmetric for the inferior implant (Fig. 3). This finding aligns Farbos De Luzan et al. (2025), which noted that pre-phonatory geometry does not necessarily predict dynamic behavior due to the complex nature of FSI.

Fig. 5c shows a strong dependence of VSG on implant vertical location. Across all implant stiffness and insertion depth conditions, VSG consistently reaches its maximum at the most inferior vertical location. As insertion depth increases, VSG generally decreases—particularly for the deepest superior insertions, where it approaches zero or even becomes negative in the case of stiffer implants. Pearson correlation coefficients between VSG and vertical location exceed 0.96 and p *<* 0.001 across all simulated conditions, indicating a universally strong positive correlation.

We further examined the relationship between VSG and three acoustic parameters—SPL, VE, and CPP—by computing Pearson correlation coefficients. VSG exhibited a strong positive correlation with SPL across all conditions within the group of interest (Fig. 5d). Similar trends were observed for VE. No consistent statistically significant correlation was observed between VSG and CPP across conditions. Overall, the correlation patterns between VSG and the acoustic outcomes closely mirrored those between vertical location and the same parameters. This alignment, along with the strong dependence of VSG on vertical location (Fig. 5c), supports the interpretation that VSG may serve as a biomechanical link through which implant vertical positioning influences acoustic output.

Our simulation results align well with the experimental findings from Oren et al. (2024) and Cohen et al. (2024), where they observed that compared to a glottal-level implant, an infraglottal level implant resulted in higher VSG, SPL and VE while no consistent improvements in CPP. We did not observe the same increase in pre-phonatory glottal closure as the implant moved from the superior to the inferior location, whereas Zhang and Chhetri (2019) attributed acoustic improvements to increased vocal fold thickness and enhanced glottal closure with inferior implantation.

### 3.4. Mechanisms for VSG-Induced improvements in SPL and VE

To further investigate the mechanism by which increased VSG improves SPL and VE, we quantified several parameters related to the energy transfer from glottal flow to the vocal folds, including the maximum divergent angle, glottal divergence height, glottal wall force asymmetry between the opening and closing phases, and the average energy transfer. The analysis was performed for the cases in the group of interest. For the maximum divergent angle and glottal divergence height, cases with convergent glottal closure are excluded.

**Fig. 6.**
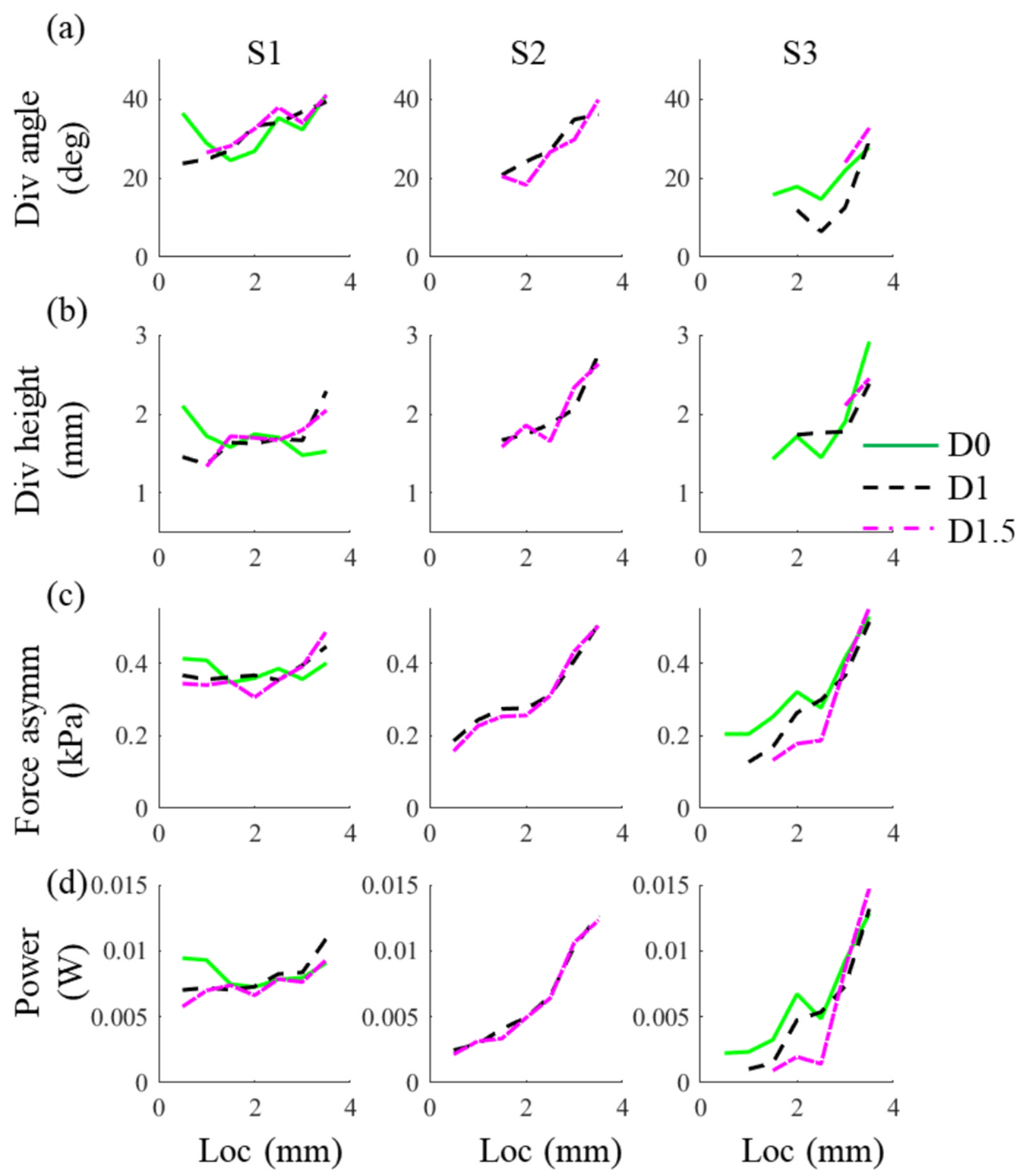
Effect of implant vertical location on flow–tissue energy transfer parameters within the group of interest: (a) Maximum divergent glottal angle; (b) Glottal divergence height; (c) Force asymmetry between the opening and closing phases; (d) Average energy transfer from glottal airflow to vocal fold tissue. Definitions of divergent angle and glottal divergence height are illustrated in the top-right subfigure of Fig. 1c. Cases with convergent glottal closure were excluded from (a) and (b).

Across most of these cases, a consistent trend emerged: shifting the implant more inferiorly produced larger maximum divergent angles (Fig. 6a), greater glottal divergence height (Fig. 6b), greater force asymmetry (Fig. 6c), and higher average energy transfer (Fig. 6d). These results provide direct evidence for the aerodynamic mechanism underlying the observed improvements in acoustic outputs and efficiency. According to the myoelastic–aerodynamic theory of phonation (Van den Berg, 1958), sustained self-oscillation of the vocal folds is driven by force asymmetry between the opening and closing phases, arising from alternating glottal shapes, allowing sustained energy transfer from airflow to vocal folds. When the implant was placed more inferiorly, the resulting larger glottal divergence height during closing (Fig. 6b) caused flow separation to occur further upstream, creating a broader low- pressure region and reducing mean wall pressure in the closing phase. The decreased wall pressure amplified the force asymmetry between opening and closing (Fig. 6c), thereby increasing the net positive energy transferred from the airflow to the vocal fold tissue (Fig. 6d). This enhanced energy exchange leads to greater vibratory amplitude, producing higher SPL and potentially contributing to the improved VE.

It should be noted that, in Bernoulli flow, flow separation is usually assumed to occur when the area ratio r ≥ 1, where r is the ratio of the area of flow separation location to the area of minimum area location. In the current study, separation is assumed at r = 1. Thus the medial surface wall pressure in the divergent part is zero, same with the fixed outlet pressure. If separation is assumed to occur at r *>* 1, flow decelerates in the divergent section. With the outlet pressure fixed at zero, it results in negative pressure in the divergent fold during the closing phase and consequently larger power transfer. The quantification of the power transfer in Fig. 6(d) thus is a conservative estimation. Moreover, the Bernoulli-based pressure estimation used in this study does not account for the effects of FSV, which would generate additional negative pressure in the divergent glottis (Oren et al., 2015, Oren et al., 2014b). Consequently, the computed mean glottal pressure depends only on glottal divergence height and omits FSV contributions. Including FSV- induced pressures would likely increase force asymmetry and energy transfer, suggesting that the effects of infraglottal implantation may be even more pronounced. Future work should employ full Navier–Stokes simulations to capture these effects.

## 4. Conclusion

We conducted a numerical parametric study to investigate the effects of infraglottal implantation—considering variations in insertion depth and implant stiffness—on a reconstructed canine laryngeal model. The results demonstrate that infraglottal implants can enhance both SPL and VE when the insertion depth exceeds a certain threshold. Under these conditions, the vertical implant location shows a strong positive correlation with SPL and VE.

Further analysis suggests that infraglottal implantation enhances VSG, which subsequently increases glottal divergence height. This leads to greater force asymmetry between the opening and closing phases, thereby promoting more effective energy transfer from the glottal airflow to the vocal folds. The result is larger vocal fold vibrations, which leads to a larger SPL and may also contribute to the improved VE. Notably, infraglottal implantation does not appear to enhance pre- phonatory vocal fold contact area.

Several limitations should be noted. First, the current simulation is based on one single canine larynx. However, the effects of implant insertion on vocal fold vibration and the resulted acoustics could vary among individuals. Future studies incorporating more subjects would help to generalize the current findings. Second, sound quality was quantified through CPP only. Incorporating addition measures, such as H1-H2 and harmonics-to-noise ratio, would provide a more comprehensive understanding of the effects of infraglottal implant on sound quality. Third, the Bernoulli flow model neglects viscous effects and requires an assumption of the flow separation location. A more accurate analysis would require Navier-Stokes equation-based flow model.

## Supplementary Material

1

## Figures and Tables

**Table 1:** The parametric space of the implant insertion. Vertical location (Loc) denotes the distance to the superior edge of the vocal fold with 0.5 mm and 3.5 mm corresponding to the most superior location and the most inferior location, respectively. Insertion depth (D) is the depth relative to a baseline value, where ~ 2.9 mm vertical height of the vocal fold contact region at the mid-coronal plane was generated with S3, Loc = 1.5 mm. A positive value indicated a deeper insertion, and a negative value indicated a shallower insertion.

Parameter	Values
Stiffness (S, kPa)	S1 (5 kPa), S2 (21 kPa), S3 (1386 kPa)
Vertical location (Loc, mm)	0.5, 1.0, 1.5, … 3.5
Insert depth relative to the baseline depth (D, mm)	−2.0, 0, 1.0, 1.5

## Appendix A. Supplementary data

Supplementary data to this article can be found online at https://doi.org/10.1016/j.jbiomech.2026.113234.
