# Supplementary material for: Effects of infraglottal implantation on voice production in type I thyroplasty: a computational study
Source: J Biomech. Author manuscript; Available in PMC 2026 Jun 23. (PMC13289833; doi:10.1016/j.jbiomech.2026.113234)
Supplement: 1 [file NIHMS2179585-supplement-1.docx]

**Supplementary: Effects of Infraglottal Implantation on Voice Production in Type I Thyroplasty: A Computational Study**

**Weili Jiang^1^, Charles Farbos de Luzan^2^, Liran Oren^2^, Ephraim Gutmark^3^, Xudong Zheng^1^ and Qian Xue^1*^**

^1^Mechanical Engineering Department, Rochester Institute of Technology, Rochester, NY 14623, USA.

^2^Department of Otolaryngology Head and Neck Surgery, University of Cincinnati, Cincinnati, OH 45267, USA.

^3^Department of Aerospace Engineering and Engineering Mechanics, University of Cincinnati, Cincinnati, OH 45267, USA

* Correspondence: [qxxeme@rit.edu](mailto:qxxeme@rit.edu)

1. **Materials and methods**

**Material property.** Both the cover and body layers of the vocal folds were modeled using a fiber-reinforced material formulation (Geng et al., 2020; Pham et al., 2018). The constitutive law of the fiber-reinforced material is Eq.1 (Dhondt, 2004):

$U=C_{10}\left( \bar{I}_{1}-3 \right)+\frac{1}{D_{1}}{(J-1)}^{2}+\frac{k_{1}}{2k_{2}}[e^{k_{2}\left( \bar{J}_{4}-1 \right)^{2}}-1]$ Eq.1

where $\bar{I}_{1}$, $\bar{J}_{4}$ are the reduced invariants of the reduced Cauchy-Green tensor, respectively; $D_{1}$ is material constant related to compressibility; $J$ is the Jacobian determinant of the deformation; $C_{10}$ is the material constant for the neo-Hooke term, and $k_{1}$, $k_{2}$ characterize the fiber reinforcement with $k_{1}$ a fiber stiffness coefficient, and $k_{2}$ a nonlinear stiffening exponent. A vertical stiffness gradient (VSG) was assumed in the cover layer, with stiffness decreasing from the inferior to superior aspect. This gradient was implemented by dividing the cover layer into six vertical bands, each assigned distinct material properties.

The material properties of the cover and body layers were optimized by matching the force-deformation relationship between numerical indentation simulations and previous experimental measurements (Michaud-Dorko et al., 2024). Numerical indentation was performed using a finite element solid mechanics solver (Dhondt and Wittig, 1998) on one side of the vocal folds discretized into 27047 tetrahedral elements. The implant was not inserted in this stage. The same solid mesh of the vocal fold and the finite element solver were subsequently used for both the virtual implantation and fluid–structure interaction (FSI) simulations. As shown in Fig S1a, a spherical indenter with a 5 mm diameter was used to indent the medial surface of the vocal fold. A contact boundary condition was defined between the surfaces of the indenter and the vocal fold. A penalization-based contact model, with a spring stiffness of 2000 Pa∙m, was applied to prevent penetration, generating a penalty force proportional to the penetration depth of the two surfaces. During numerical indentation, force was applied on the indenter to push it into the vocal fold, and the vocal fold displacement was recorded. The indentation was performed at six different locations on the vocal fold surface (Fig S1a). At each location, four indentation forces were simulated, and the genetic-algorithm-based optimization was used to determine material parameters by minimizing the difference between numerically predicted and experimentally measured displacement. The optimized force-displacement curves are compared to experimental data in Fig S1b, showing strong agreement across all locations. The simulation results closely matched the experimental data at the center and posterior locations, with an error of 5.4% ± 5.0% (mean ± standard deviation). At the anterior, the simulation predicted a softer response in the superior region and a stiffer response in the inferior region compared to the experiment, resulting in an error of 11.3% ± 3.2%. The final optimized material parameters are summarized in Table SI and the same material parameters were employed in both sides of the vocal folds.

The Neo-Hookean material model was used to describe the material properties of all cartilages and the paraglottic space. The corresponding constitutive law is given in Equation 2.

$U=C_{10}\left( \bar{I}_{1}-3 \right)+\frac{1}{D_{1}}{(J-1)}^{2}$ Eq.2

The material parameters were chosen to match the reported values in the literature. The paraglottic space was assumed to be composed of adipose (fat) tissue (Wu and Zhang, 2021). The final material parameters are summarized in Table S1.


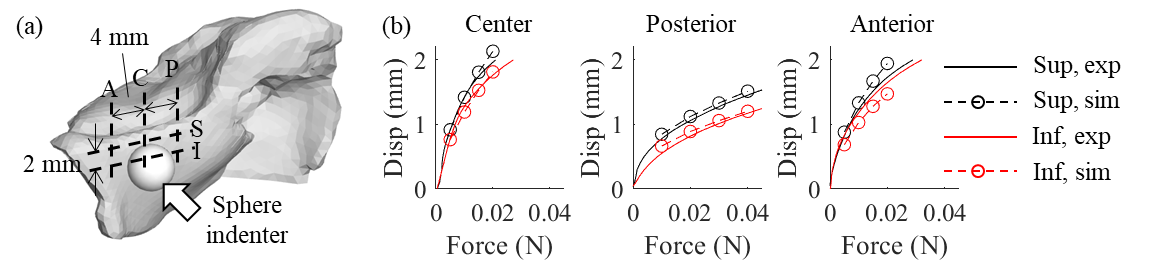


**Fig S1.** Numerical indentation for obtaining the material parameters. (a) A sphere indenter was pushed into the tissue at six locations across the medial surface. Superior and inferior locations were 2 mm apart. Anterior, center and posterior locations were 4 mm apart. (b) The comparison of the force-displacement curves between the experiment (Michaud-Dorko et al., 2024) and simulation results.

**Table SI**. Parameters of the material model (Dhondt, 2004; Geng et al., 2020; Jiang et al., 2024).

|  | Constitutive law | Model parameters (kPa) |
| --- | --- | --- |
| Vocal folds - body | Fiber-reinforced model | $C_{10}$=0.45, $k_{1}$=1.42, $k_{2}$=8.87 |
| Vocal folds - cover | Fiber-reinforced model | $C_{10}$ changes from 0.37 to 0.29 from inferior to superior; $k_{1}$=1.15, $k_{2}$ =5.47 |
| Cartilages | Neo-Hooke | $C_{10}$=5000 |
| Paraglottic space | Neo-Hooke | $C_{10}$=0.41 |

**Virtual implantation.** A virtual implantation was performed on the paralyzed (left) vocal fold to generate the pre-phonatory posture. The implant was modeled as a Neo-Hookean material and discretized using 6864 tetrahedron elements. It was inserted through a virtual rectangular window measuring 5 × 10 mm, oriented parallel to the inferior border of the thyroid cartilage. The window’s anterior edge was located about 5 mm posterior to the anterior margin of the thyroid cartilage (Netterville et al., 1993; Rapoport and Courey, 2022) and inferior edge was varied along vertical direction. To simulate the insertion process, a displacement boundary condition was applied at the base of the implant, pushing it into the paraglottic space in the transverse plane. The lateral surface of the paraglottic adipose layer was assumed to be detached from the thyroid cartilage, enabling realistic deformation of the vocal fold in response to the implant insertion. A contact boundary condition was defined between the medial surface of the implant and the lateral surface of the paraglottic layer to capture the interaction and stress redistribution during medialization. Still, penalization-based contact model was applied to prevent penetration. In addition, several nodes along the superior edge of the paraglottic layer were fixed to mimic the surgical sutures used in the experiments. The detailed methodology of the virtual implantation procedure is described in our previous work (Movahhedi et al., 2021).

During medialization, the cricoid cartilage was fixed in place. The translational motion of the thyroid cartilage at the cricothyroid joint (CTJ) was restricted, while rotational motion about the CTJ was allowed. The arytenoid cartilage was permitted to slide and rotate around a predefined axis representing the cricoarytenoid joint (CAJ). Further details on the CTJ and CAJ constraints are provided in (Geng et al., 2020). Medialization of the healthy side was achieved by applying a medial point force to its vocal process.

**FSI simulation.** Following virtual implantation, FSI simulations were conducted using the deformed vocal fold and implant. All other surfaces connected to surrounding cartilages were assigned fixed boundary conditions based on their deformed configurations from the virtual implantation. This approach preserves the realistic pre-phonatory geometry and internal stress distribution while significantly reducing computational cost by eliminating the need to explicitly model the cartilages.

To simulate FSI, the finite element model of the vocal fold and implant was coupled with a one-dimensional reduced-order glottal flow model based on the Bernoulli equation (Geng et al., 2021), providing a computationally efficient solution required for the parametric study, though it neglects viscous effects. The FSI interface was defined along the medial and inferior surfaces of the vocal fold, where pressure loads were computed by the fluid solver and transferred to the solid solver as nodal forces. The resulting tissue deformation was then used to update the cross-sectional area of the flow channel. A constant subglottal pressure of 1.5 kPa (Farbos De Luzan et al., 2025; Oren et al., 2024) was applied in all cases. Penalization-based contact model was defined between the medial surfaces of the healthy and paralyzed vocal folds.

In the reduced-order glottal flow model, Bernoulli’s equation was applied from the subglottal region up to the location of the minimum glottal area, where flow separation was assumed to occur. Beyond this point, in the divergent segment of the glottis, the pressure was assumed to drop to ambient (zero-gauge pressure) due to separation and jet formation. Under these assumptions, the glottal flow rate ($Q$) was calculated as:

$Q=A_{min}\sqrt{\frac{2p_{sub}}{\rho}}$ Eq.3

where $A_{min}$​ is the minimum cross-sectional area of the glottis, $p_{sub}$ is the subglottal pressure, and $\rho$ = 1 kg/m^3^ is the air density.

The pressure distribution $p\left( y \right)$ along the glottis was computed only in the converging section leading up to the separation point (up to the location of $A_{min}$​​) using:

$p\left( y \right)=p_{sub}-\frac{1}{2}\rho{(\frac{Q}{A(y)})}^{2}$ Eq.4

where $A\left( y \right)$ is the local cross-sectional area at position $y$. Downstream of $A_{min}$​, the pressure was set to zero gauge.

**Parametric setup.** The three stiffness values, including 5 kPa, 21 kPa, and 1386 kPa, are consistent with previous studies (Reddy et al., 2022; Wu and Zhang, 2023). Specifically, the 1386 kPa value corresponds to medical-grade Silastic, while the 5 kPa and 21 kPa implants were achieved by mixing silicone solutions at different ratios. Four insertion depths were evaluated relative to a baseline depth, which was defined as the depth that produced approximately a vertical height of the 2.9 mm of the vocal fold contact surface, measured at the mid-coronal plane for the 1386 kPa implant and Loc = 1.5 mm. Positive and negative values represent insertions deeper or shallower than this baseline, respectively. In the superior–inferior direction, seven vertical implant locations were tested, ranging from 0.5 mm to 3.5 mm below the superior edge of the vocal fold (Fig. 1d). The 0.5 mm and 3.5 mm locations correspond to the glottal level and infraglottal level, respectively, following the terminology used in Farbos De Luzan et al. (2025) and Oren et al. (2024).

**Data analysis**.

Sound pressure ($p^{'}$) was calculated using Eq.5 (Lighthill, 1978):

$p^{'}=\frac{\rho}{4\pi r}\frac{dQ}{dt}$ Eq.5

where $r$ is the distance from the source, assuming to be 0.3 m in the current study. $\rho$ = 1.0 kg/m^3^ is the air density.

Sound pressure level (SPL) was calculated as Eq.6:

$SPL=20{log}_{10}\frac{p_{rms}}{p_{0}}$ Eq.6

where $p_{rms}$ is the root-mean-square value of the sound pressure and $p_{0}$ = 2×10^-5^ Pa, is the reference pressure.

Cepstral peak prominence (CPP) was calculated using Praat (Boersma and Weenink, 2025; Heller Murray et al., 2022) based on $p^{'}$. CPP is defined as the amplitude difference between the first harmonic peak and the base regression line, reflecting the overall level of noise in the sound. The parameter setting in Praat for calculating CPP follows the ones in Heller Murray et al. (Heller Murray et al., 2022).

Vocal efficiency (VE) was calculated as the ratio of the acoustic power to aerodynamic power (Eq.7):

$VE=\frac{P_{acou}}{P_{aero}}=\frac{Ap^{2}/\rho c}{p_{sub}Q}=\frac{4\pi r^{2}{p_{rms}}^{2}/\rho c}{p_{sub}Q_{mean}}$ Eq.7

where *c* = 340 m/s is the sound speed and $Q_{mean}$ is the mean glottal flow rate. The calculation of the aerodynamic power was based on Titze (Titze, 1992). The calculation of the acoustic power assumed that the acoustic pressure propagates spherically.

Key statistical quantities related to the vocal fold and glottal flow dynamics were computed for analysis. The glottal area ($A_{min}$) at each time instant is the minimum cross-sectional area of the glottis. The maximum glottal area is defined as the peak glottal area in a vibration cycle. $y_{A, min}$ is the axial location corresponding to $A_{min}$. Four marker points were defined in the mid-coronal plane corresponding to the superior edge (n1 and n2) of the vocal folds and $y_{A, min}$ (n3 and n4). The axial distance between the axial location of n1, n2 and $y_{A, min}$ is defined as the glottal divergence height ($h$). The glottal angle ($\alpha$) was defined as the angle formed by two straight lines connecting n1, n3 and n2, n4, respectively. The MFDR was determined by measuring the peak negative value of the flow changing rate ($\frac{dQ}{dt}$), which occurred in the closing phase. Absolute value was used later in the analysis. The closed quotient was defined as the ratio of the duration of closed glottis to the fundamental period. The skewness quotient was defined as the ratio of the duration of flow rate increase to that of decrease. The maximum divergent angle represents the largest glottal angle observed during the closing phase. The glottal divergence height was defined as the vertical distance from the superior edge of the vocal fold at the mid-coronal plane to the location of minimum cross-sectional area during closing phase. Force asymmetry was quantified as the difference between the maximum wall pressure during the opening phase and the minimum wall pressure during the closing phase, where the wall pressure in each time instant was defined as the spatially averaged pressure of the medial and inferior vocal fold surfaces. The energy transfer was quantified using Eq.8:

$P_{j}=\sum_{i=1}^{n} p_{i}\cdot A_{i}\cdot\hat{n}_{i}\cdot v_{i}$

$E=\sum_{t=t_{1}}^{t_{2}} \left( dt\cdot P_{j} \right)/(t_{2}-t_{1})$ Eq.8

where $P_{j}$ is the power transferred from the glottal flow to the vocal fold at the *j* th time instant; *i* loops the mesh elements on the FSI interface; $p_{i}$, $A_{i}$, $\hat{n}_{i}$, $v_{i}$ are the pressure, area, face normal, and velocity of the *i* th element, respectively; $P_{j}$ in discrete time instants were integrated in the time duration between $t_{1}$ and $t_{2}$ and then averaged by dividing the time period ($t_{2}-t_{1}$) to obtain the averaged energy transfer *E*. The average values across multiple cycles were computed for the above parameters, excluding the transition phase at the beginning of the simulation.

To assess the effect of vocal fold adduction, we defined the pre-phonatory contact area as the integrated area along the vocal folds where the inter-fold distance was below 0.01 mm, representing regions of near contact used to approximate tissue collision or closure. To quantify the pre-phonatory VSG, numerical indentation was performed at two vertical locations in the mid-coronal plane on the paralyzed fold with implant inserted. A lateral force, *F*, was applied, and the resulting lateral displacement of the indenter, *x*, was recorded at both the superior and inferior locations, denoted as $x_{sup}$ and $x_{inf}$, respectively. These two locations were spaced 4 mm apart, with the superior location positioned just below the superior edge of the vocal fold. The VSG was then calculated using the following formula:

$VSG=\frac{k_{inf}-k_{sup}}{k_{inf}+k_{sup}}=\frac{F/x_{inf}-F/x_{sup}}{F/x_{inf}+F/x_{sup}}=\frac{x_{sup}-x_{inf}}{x_{inf}+x_{sup}}$ Eq.9

where $k=F/x$ represents the stiffness measured from indentation for different locations.

Pearson correlation coefficient – $\rho\left( X,Y \right)$, denoted using r – was computed to quantify the linear correlation between different parameters using Eq.10:

$\rho\left( X,Y \right)=\frac{cov(X,Y)}{\sigma_{X}\sigma_{Y}}$ Eq.10

where $X$ and $Y$ are the two variables, $cov(X,Y)$ is the covariance of $X$ and $Y$, and $\sigma_{X}$ and $\sigma_{Y}$ are the standard deviation of $X$ and $Y$, respectively. The coefficient r ranges from -1 to 1, indicating both the strength and direction of the correlation. An r value of 1 or -1 represents a perfect positive or negative linear correlation, respectively, meaning that the two variables increase or decrease together, or vary in opposite directions. Correlations with |r| >0.7 were classified as strong; correlations with 0.5<|r| ≤ 0.7 were classified as moderate; correlations with 0.3<|r| ≤ 0.5 were classified as weak; correlations with |r|≤0.3 were classified as negligible (Mukaka, 2012). Correlations were considered statistically significant when *p*-value was less than 0.05.

1. **Results and discussion**

**Additional comments about Figure 5a.** Strong negative correlations (*r* < –0.86, *p* < 0.014) between vertical location and pre-phonatory contact area were observed in the D = 0.0mm, S1 condition and across all stiffness levels with D = -2.0 mm. Even in these cases, however, the maximum reduction in pre-phonatory contact area as the implant shifted from the superior to the inferior location was only 16% (S3, D = –2.0 mm).

**Additional comments about Figure 6.** Among all the cases presented in Fig. 6, the S1 case with D = 0.0 mm did not exhibit a clear positive correlation with vertical implant location. No consistent trend was observed across the four quantified parameters shown in Fig. 6. Detailed analysis of the vibration patterns revealed a shift in vibratory behavior within this group. For Loc = 0.5 mm and 1.0 mm, the vibration was approximately symmetric between the left and right vocal folds, with pronounced vertical motion. In contrast, the remaining cases exhibited significantly reduced vertical motion and strong left-right asymmetry. This shift in vibration mode likely contributed to the non-monotonic trends observed in Fig. 6. It is also important to note that in several cases – the divergent angle and divergence height in S3 (Fig. 6a and b) and the force asymmetry in S1 for D = 1.0 mm and 1.5 mm conditions (Fig. 6c) – though the Pearson correlation coefficient suggested a strong correlation (r>0.7), the p-value exceeded 0.05. Since p-value signifies the probability of committing a Type I error, it indicates that more data points are needed to confirm this strong correlation. Nevertheless, the overall trend across the full dataset still indicated a positive correlation between vertical implant location and the quantified parameters, despite local deviations in certain cases.

**Reference**

Boersma, P., Weenink, D., 2025. Praat : doing phonetics by computer [Computer program].

Dhondt, G., 2004. The Finite Element Method for Three‐Dimensional Thermomechanical Applications. Wiley.

Dhondt, G., Wittig, K., 1998. A Free Software Three-Dimensional Structural Finite Element Program [WWW Document]. URL http://www.dhondt.de/

Farbos De Luzan, C., Michaud-Dorko, J., Howell, R.J., Gutmark, E., Oren, L., 2025. Dynamically Quantifying Vocal Fold Thickness: Effects of Medialization Implant Location on Glottal Shape and Phonation. Bioengineering 12, 667. https://doi.org/10.3390/bioengineering12060667

Geng, B., Movahhedi, M., Xue, Q., Zheng, X., 2021. Vocal fold vibration mode changes due to cricothyroid and thyroarytenoid muscle interaction in a three-dimensional model of the canine larynx. J. Acoust. Soc. Am. 150, 1176–1187. https://doi.org/10.1121/10.0005883

Geng, B., Pham, N., Xue, Q., Zheng, X., 2020. A three-dimensional vocal fold posturing model based on muscle mechanics and magnetic resonance imaging of a canine larynx. J. Acoust. Soc. Am. 147, 2597–2608. https://doi.org/10.1121/10.0001093

Heller Murray, E.S., Chao, A., Colletti, L., 2022. A Practical Guide to Calculating Cepstral Peak Prominence in Praat. J. Voice S0892199722002752. https://doi.org/10.1016/j.jvoice.2022.09.002

Jiang, W., Geng, B., Zheng, X., Xue, Q., 2024. A computational study of the influence of thyroarytenoid and cricothyroid muscle interaction on vocal fold dynamics in an MRI-based human laryngeal model. Biomech. Model. Mechanobiol. 23, 1801–1813. https://doi.org/10.1007/s10237-024-01869-9

Lighthill, M., 1978. Waves in fluids. Cambridge University Press, Cambridge, MA.

Michaud-Dorko, J., Dion, G.R., Farbos De Luzan, C., Gutmark, E., Oren, L., 2024. Characterization of the Vertical Stiffness Gradient in Cadaveric Human and Excised Canine Larynges. J. Voice S0892199724002601. https://doi.org/10.1016/j.jvoice.2024.08.011

Movahhedi, M., Geng, B., Xue, Q., Zheng, X., 2021. A computational framework for patient-specific surgical planning of type 1 thyroplasty. JASA Express Lett. 1. https://doi.org/10.1121/10.0009084

Mukaka, M.M., 2012. Statistics corner: A guide to appropriate use of correlation coefficient in medical research. Malawi Med. J. 24.

Netterville, J.L., Stone, R.E., Civantos, F.J., Luken, E.S., Ossoff, R.H., 1993. Silastic Medialization and Arytenoid Adduction: The Vanderbilt Experience: A Review of 116 Phonosurgical Procedures. Ann. Otol. Rhinol. Laryngol. 102, 413–424. https://doi.org/10.1177/000348949310200602

Oren, L., Maddox, A., Farbos De Luzan, C., Xie, C., Howell, R., Dion, G., Gutmark, E., Khosla, S., 2024. Acoustics and aerodynamic effects following glottal and infraglottal medialization in an excised larynx model. Eur. Arch. Otorhinolaryngol. 281, 2523–2529. https://doi.org/10.1007/s00405-024-08519-x

Pham, N., Xue, Q., Zheng, X., 2018. Coupling between a fiber-reinforced model and a Hill-based contractile model for passive and active tissue properties of laryngeal muscles: A finite element study. J. Acoust. Soc. Am. 144, EL248–EL253. https://doi.org/10.1121/1.5055564

Rapoport, S.K., Courey, M.S., 2022. Type I Thyroplasty and Arytenoid Adduction: Review of the Literature and Current Clinical Practice. Int. J. Head Neck Surg. 12, 166–171. https://doi.org/10.5005/jp-journals-10001-1517

Reddy, N., Lee, Y., Zhang, Z., Chhetri, D.K., 2022. Optimal thyroplasty implant shape and stiffness for treatment of acute unilateral vocal fold paralysis: Evidence from a canine in vivo phonation model, in: Interspeech 2022. ISCA, ISCA, pp. 2273–2277. https://doi.org/10.21437/Interspeech.2022-11158

Titze, I.R., 1992. Vocal efficiency. J. Voice 6, 135–138. https://doi.org/10.1016/S0892-1997(05)80127-4

Wu, L., Zhang, Z., 2023. Effects of implant and vocal fold stiffness on voice production after medialization laryngoplasty in an MRI-based vocal fold model. J. Biomech. 149, 111483. https://doi.org/10.1016/j.jbiomech.2023.111483

Wu, L., Zhang, Z., 2021. Impact of the Paraglottic Space on Voice Production in an MRI-Based Vocal Fold Model. J. Voice. https://doi.org/10.1016/j.jvoice.2021.02.021
